# Gene Expression Analysis of PTEN Positive Glioblastoma Stem Cells Identifies DUB3 and Wee1 Modulation in a Cell Differentiation Model

**DOI:** 10.1371/journal.pone.0081432

**Published:** 2013-12-12

**Authors:** Stefano Forte, Alfredo Pagliuca, Eugenia T. Maniscalchi, Rosario Gulino, Giovanna Calabrese, Lucia Ricci-Vitiani, Roberto Pallini, Michele Signore, Rosalba Parenti, Ruggero De Maria, Massimo Gulisano

**Affiliations:** 1 IOM Ricerca srl, Viagrande, Italy; 2 Department of Hematology, Oncology and Molecular Medicine, Istituto Superiore di Sanità, Rome, Italy; 3 Institute of Neurosurgery, Catholic University School of Medicine, Rome, Italy; 4 Department of Biomedical Sciences, University of Catania, Italy; 5 Italian National Cancer Institute ‘Regina Elena’, Rome, Italy; University of Catania, Italy

## Abstract

The term astrocytoma defines a quite heterogeneous group of neoplastic diseases that collectively represent the most frequent brain tumors in humans. Among them, glioblastoma multiforme represents the most malignant form and its associated prognosis is one of the poorest among tumors of the central nervous system. It has been demonstrated that a small population of tumor cells, isolated from the brain neoplastic tissue, can reproduce the parental tumor when transplanted in immunodeficient mouse. These tumor initiating cells are supposed to be involved in cancer development and progression and possess stem cell-like features; like their normal counterpart, these cells remain quiescent until they are committed to differentiation. Many studies have shown that the role of the tumor suppressor protein PTEN in cell cycle progression is fundamental for tumor dynamics: in low grade gliomas, PTEN contributes to maintain cells in G1 while the loss of its activity is frequently observed in high grade gliomas. The mechanisms underlying the above described PTEN activity have been studied in many tumors, but those involved in the maintenance of tumor initiating cells quiescence remain to be investigated in more detail. The aim of the present study is to shed light on the role of PTEN pathway on cell cycle regulation in Glioblastoma stem cells, through a cell differentiation model. Our results suggest the existence of a molecular mechanism, that involves DUB3 and WEE1 gene products in the regulation of Cdc25a, as functional effector of the PTEN/Akt pathway.

## Introduction

Glioblastoma multiforme (GBM) is the most malignant form of glioma and its associated prognosis is one of the poorest among tumors of the central nervous system (CNS). It has been recently demonstrated that a small population of tumor cells, with stem cell-like features (GSCs), can initiate tumor development *in vivo* and are thought to be involved in tumor maintenance and diffusion [1], [2]. These cells, as well as their normal counterparts, tightly regulate their growth while uncommitted but quickly gain uncontrolled proliferation as soon as they are committed to differentiate. Molecular switches underlying these phenomena are tightly linked to the features of disease development and progression.

It has been shown that the tumor suppressor protein PTEN is involved in cell cycle regulation, through the modulation of different signaling pathways, either in cancer in general and in brain tumor in particular [3], [4], [5]. The functional role of PTEN in brain cancers progression seems to be tightly linked to tumor dynamics: in low grade gliomas, PTEN contribute to maintain cells in G1 while the loss of its activity is frequently observed in high grade gliomas [4]. One of the most characterized downstream effectors of PTEN is Akt [6]. In particular, many studies investigating the PTEN-dependent cell cycle regulation, reported the the influence of PTEN/Akt signaling pathway on post-translational activation and stability of cell cycle regulatory proteins [7], [8]. To identify downstream molecular actors involved in PTEN mediated cell cycle modulation, a prostatic cancer cellular model expressing PTEN cDNA was recently used [9]. This modification led to a significant inhibition of cell proliferation due to cycle arrest in the G1 phase. Gene expression analysis subsequent to PTEN reintroduction, coupled with phosphorylation status of downstream molecular targets, suggest that PTEN physiologically regulates cell cycle related proteins, through pAKT dependent and independent ways. E2F2, cdc25a, Cyclin G2 and RBL2 proteins are among them [9], [10].

The aim of the present study was to shed light on the role of PTEN pathway on cell cycle regulation in GSCs through a cellular differentiation model. Phosphoproteomics profiling of different GSCs lines, using reverse phase protein microarrays (RPMA), indicates a heterogeneous basal activity of PTEN and related molecules. Transcriptomics profiling of GSCs induced to differentiate demonstrates specific molecular changes in PTEN positive cells, that are not observed in cells lacking its activation. The most modulated transcripts suggest a post-transcriptional regulation model, which involves cdc25a as the main target.

## Materials and Methods

### Ethics statement

This study has been approved by Institute of Neurosurgery, Catholic University School of Medicine, Rome, Italy. Before surgery patients provided written consent.

### Patients, diagnosis, and tumor characterization

Tumor tissue samples were collected from 8 patients with primitive brain tumor undergoing complete or partial surgical resection at the Institute of Neurosurgery, Catholic University School of Medicine, in Rome, Italy. A diagnosis of glioblastoma multiforme, established histologically according to the WHO classification [11], was the eligibility criteria. Patients characteristics are summarized in table 1. Tumor samples were obtained by complete or partial resection before the initiation of treatment with radiation and chemotherapy. The expression of both the proliferation marker Ki-67 and of tumor protein 53 (P53) were characterized on tumor specimen by immunohistochemistry on deparaffinized sections using the avidin-biotin-peroxidase complex methods (ABC-Elite kit, Vector Laboratories, anti-Ki67 monoclonal antibody (MIB-1, Dako), anti-p53 monoclonal antibody (DO-7, Dako) and anti-epidermal growth factor receptor (EGFR) monoclonal antibody (EGFR.25, Novocastra). Tumors were considered p53 deficient if immunoreaction stained the nuclei of less than 5% of cells [12], [13]. Tumors showing moderate-to-strong immunostaining for EGFR in >20% of cells were considered EGFR positive [14]. O6-methylguanine-DNA methyltransferase (MGMT) promoter methylation patterns were assessed on genomic DNA extracted from paraffin-embedded tissue by methylation-specific: it has been shown that the DNA repair protein MGMT influence the resistance of glioblastoma cells to alkylating agents, such as nitrosoureas and temozolomide thus representing and important prognostic and therapeutic indicator [15].

**Table 1:** Patients characteristics.

Sample	sex	Age (yr)	KPS (score)	Symp. (mo.)	Location	ki 67 (%)	p53	MGMT	EGFR	PFS	OS	PTEN activ
a	**M**	**40**	**80**	**2.5**	**Temporal**	**20**	**+**	**+**	**+**	**6.0**	**12.5**	**low**
b	**M**	**77**	**80**	**2**	**Parietal**	**50**	**+**	**−**	**+**	**1.0**	**2.0**	**high**
c	**M**	**72**	**90**	**1.5**	**Frontal**	**5**	**−**	**+**	**−**	**6.0**	**11.5**	**high**
d	**M**	**44**	**80**	**1**	**Frontal**	**10**	**−**	**+**	**+**	**5.0**	**7.5**	**low**
e	**M**	**59**	**80**	**2**	**Occipital**	**35**	**+**	**−**	**+**	**3.0**	**6.0**	**low**
f	**M**	**48**	**60**	**2.5**	**Parietal**	**20**	**+**	**−**	**+**	**1.0**	**2.0**	**high**
g	**M**	**58**	**70**	**3**	**Parietal**	**10**	**−**	**−**	**−**	**4.0**	**10.5**	**high**
h	**M**	**52**	**70**	**0.5**	**Temporal**	**40**	**+**	**−**	**+**	**3.0**	**8.0**	**high**

=  sample identifier, sex  =  patient sex, KPS (score)  =  Karnofsky Performance Status score; Sympt. (mo)  =  symptom duration in month; Surgery (type)  =  origin of tumor tissue from the patient brain (temporal/parietal/occipital/frontal); ki67-%  = % of cells expressing ki-67; p53 =  p53 positivity (less than 5% of nuclei); MGMT  =  MGMT promoter metylation; EGFR  =  EGFR positivity (moderate-to-strong signal on >% of cells); PFS  =  progression-free survival; OS  =  overall survival; PTEN activ  =  PTEN activation group according to clustering of phospoproteomics profiles. Characteristics of patients from which samples were collected. Legend: Sample

### Cell culture and neural differentiation

GSCs were obtained from freshly dissociated surgical specimens cultivated in a serum-free medium containing epidermal growth factor (EGF) and basic fibroblast growth factor (FGF), as previously described [16], [17], [18]. In these conditions, cells grow as clusters of undifferentiated cells (neurospheres). For the differentiation protocol, 5×10^5^ cells were plated in matrigel (1∶25 in stem cell medium) coated flasks and induced by growth factors deprivation and supply of 5% serum in the growth environment. After 14 days, cells were checked for differentiation markers by flow cytometry and for cellular morphology. Cytofluorimetric analysis was performed using a FACS Canto flow cytometer (Becton Dickinson) to assess the expression of CD133, Sox2 and nestin (NES), as stem cells markers, and glial fibrillary acidic protein (GFAP) and beta 3 tubulin (TUBB3), as differentiation markers (Miltenyi Biotec antibodies). Cellular immunophenotype was also assessed by means of a laser confocal microscope (Leica TCS SP5, Leica Microsystem S.p.A.). Primary antibodies against Sox2, GFAP (both from Cell Signaling Technology) and TUBB3 (Millipore) were diluted in 1% BSA to a final concentration of 5 μg/ml and incubated for 1 h.

### Reverse phase protein arrays

RPPA was performed as previously described [19]. Samples were lysed using Tissue Protein Extraction Reagent (Thermo Scientific, Waltham, MA, USA) and diluted up to 0.5 mg/mL with Novex Tris-Glycine SDS Sample Buffer 2X (Invitrogen Corporation, Carlsbad, CA, USA). Each lysate was spotted in a two-fold 5 point dilution curve onto nitrocellulose-coated microscope slides using an Aushon Arrayer 2470 (Billerica, MA, USA) and slides underwent incubation with a single validated primary antibody using DAKO Autostainer Plus (DAKO Corporation, Glostrup, Denmark). Total protein quantification was performed using Sypro Ruby Protein Staining solution (Invitrogen) and slides were scanned using a Vidar Revolution 4200 microarray scanner (Vidar Systems Corporation, Herndon, VA, USA). Antibody slides were scanned using a flatbed scanner and raw images were processed for secondary antibody subtraction and normalization to total protein.

### Gene expression profiling

Samples transcriptomes were characterized using Affymetrix GeneChip Human Gene 1.0 ST arrays. Total RNA was extracted and processed according to manufacturer procedures. CEL files were quality checked, acquired and processed using Affymetrix Power Tools with RMA normalization option. Preprocessed data are available as file S1.

### Statistical analysis and functional annotations

PTEN positive and PTEN negative groups of samples were defined using unsupervised hierarchical clustering of three dimensional (PTEN, AKT and pAKT) protein expression profiles. Euclidean metric was used for distance calculations. Differences in gene expression profiles were evaluated using paired t test (95% confidence intervals, α = 0.05). Association between gene expression levels of candidate transcripts and PFS or OS were assessed using a Cox proportional hazards regression model. Clustering, plots and t statistics were produced using R statistical environment [20].

## Results

### Differentiation

14 days after induction, differentiated cells show glial fibrillary acidic protein (GFAP) and beta 3 tubulin (TUBB3), while they lose the expression of CD133, Sox2 and nestin (NES) when assessed by cytoflourimetry. During the induction, cells proliferate to reach a count of 2,5 millions on average.

**Figure 1:**
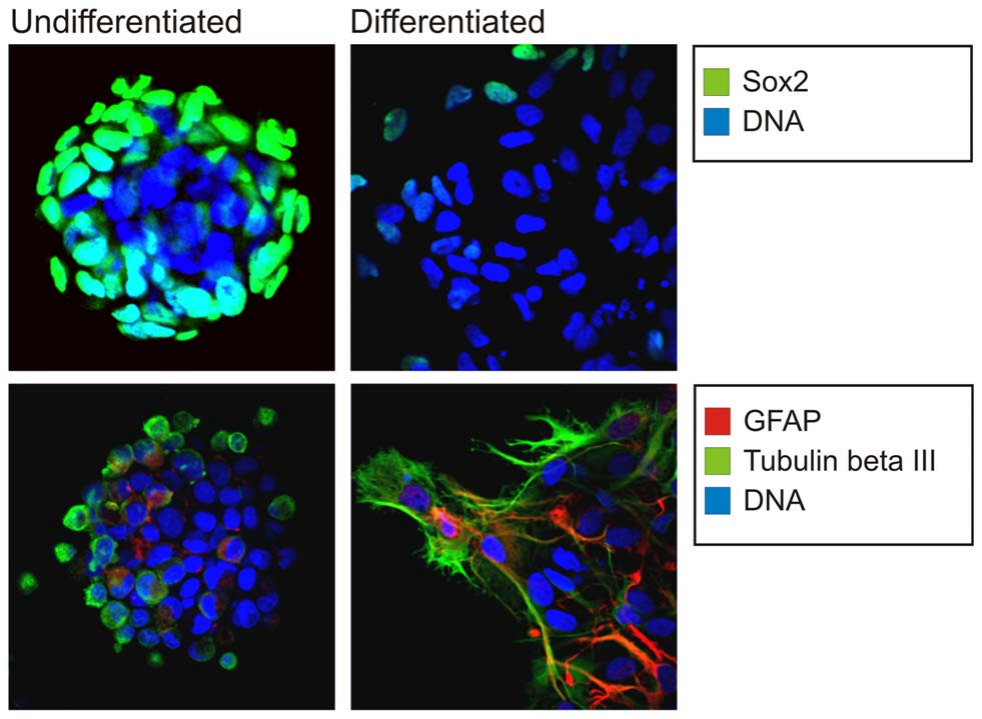
Immunophenotype of undifferentiated and differentiated cells. Immunofluorescence of undifferentiated GSCs neurosphere (left panels) and of differentiated cells (right panels) labelled with anti-human Sox2 (top panels), anti-human GFAP and anti-human TUBB3 (bottom panels).

Neurospheres show the expression of Sox2 while differentiated cells, which lack its expression, show the expression of the differentiation markers GFAP and TUBB 3 (Figure 1).

### Phosphoproteomics

**Figure 2:**
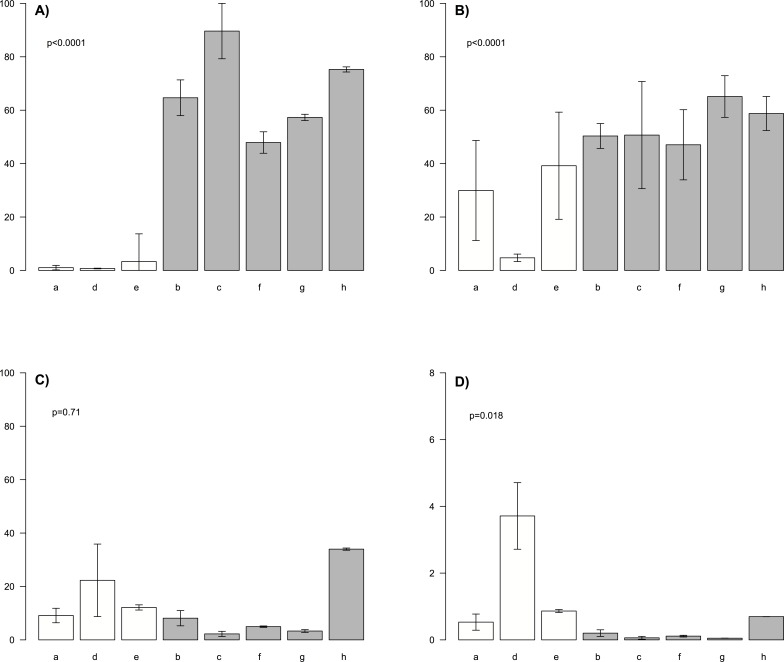
Protein expression profiles of GSC samples. (A) PTEN, (B) AKT, (C) pAKT and (D) pAKT/AKT proteins expression profiles in different lines of GSCs. Bars are coloured according to groups previously defined using hierarchical clustering of protein profiles: white bars are used for PTEN negative cell lines while grey bars for PTEN positive.

The analysis of phosphoproteomics profiles shows that GSCs may present differences, in basal conditions, in the activation status of PTEN pathway. Its global activity has been checked by measuring the protein expression levels of PTEN and of its downstream molecular partner AKT, which behaviour can be regulated by specific phosphorylation events. In particular, we analyzed the presence of activatory S473 phosphoepitope of AKT. Three cell lines (a, d and e) showed proteins profiles which are related to the lack of PTEN pathway activity (low PTEN and positive signal for the active phosphothreonine AKT form) while all the other lines showed profiles which indicate PTEN pathway activation (figure S1). According to these observation PTEN positive and PTEN negative cell lines were defined (Figure 2).

**Figure 3:**
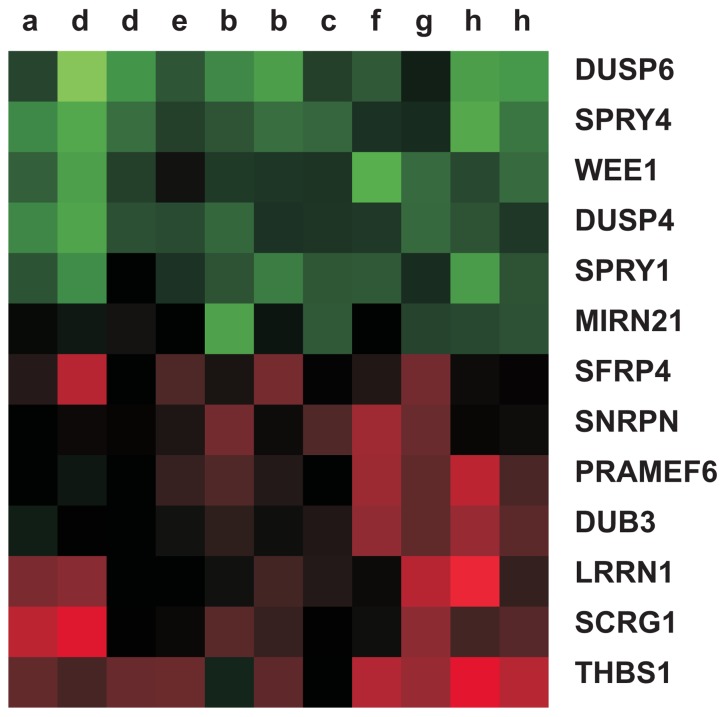
Transcripts fold change in differentiation. Green, red and black indicate transcripts that decrease, increase or maintain their concentration during induction respectively.

Samples were grouped according to the above-described molecular criterion and cells were induced to differentiate. Gene expression profiles in both groups were analyzed comparing differentiated and undifferentiated samples, in order to identify transcripts that are specifically modulated during differentiation. Profiles were analyzed comparing each differentiated sample with its corresponding baseline; then, transcripts that showed a statistically significant modulation in a paired test were selected. Transcripts having less than 2 fold of modulation in logarithmic scale in more than 30% of samples were discarded because the overall intensity of variation wasn't considered relevant in sample population. Results are presented in figure 3. In general, cells induced to differentiate show a positive regulation of deubiquitinating enzyme 3 (DUB3), thrombospondin 1 (THBS1), Leucine Rich repeat neuronal 1 (LRRN1) and Secreted Frizzled related protein 4 (SFRP4) transcripts and a negative regulation of Wee1, miR 21, Sprouty homolog 4 (SPRY4), Sprouty homolog 1 (SPRY1), Dual specificity phosphatase 6 (DUPS6), Dual specificity phosphatase 4 (DUSP4) ones.

These transcripts showed no significant associations between expression levels and patients or specimen characteristics (location, surgery type, ki 67%, p53 positivity, MGMT, EGFR, PFS, OS).

### Differences in PTEN active and inactive groups

When PTEN active and inactive cell lines are compared, differences are observed in the extent of modulation observed in previously selected transcripts. While Dub3 and mir21 are significantly modulated (with upregulation and downmodulation respectively) in PTEN active cell lines, their transcripts variation is far less evident than in PTEN inactive ones. Wee1 is significantly downmodulated in both groups.

## Discussion

Stem cells tightly regulate cell cycle progression in order to remain quiescent inside their cellular niche with progress occurring only when external stimuli indicates the need for cell growth or for the replacement of damaged or senescent cells. This process is so crucial that involved molecules are subjected to multiple levels of regulation. Cell-cycle transitions can be often the results of coordinate factors which involves rapid turnover of crucial regulatory proteins through the ubiquitin-proteasome system. These mechanisms are shown to be frequently altered when observing cancer cell proteomes, and dysregulation of these pathways is clearly linked to the acquisition of neoplastic phenotype.

A paradigmatic example is represented by the cell division cycle 25A (Cdc25A) protein, which is often found expressed in high concentration in many cancers [21], [22], [23]. No genetic amplification events or stabilizing mutations have been reported in literature for Cdc25a, suggesting that its modulation in tumors could be due to different regulatory mechanisms. Pereg and colleagues [24] defined a simple circuit of Cdc25a post translational regulation in breast cancer cell, starting from the observation that there is no significant correlation between Cdc25A mRNA and protein levels [25]. According to this model even if Cdc25a mRNA is constantly expressed during each of the cell-cycle stages, its protein level is kept low during G1 by proteasomal degradation that follows the ubiquitination operated by APC protein. DUB3 acts as an inhibitor of Cdc25a ubiquitination thus providing stability to it. DNA damage checkpoints proteins contribute to Cdc25 protein modulation, during S phase, though the activation of SCF^β-TRCP^-dependent turnover (which also rely on Cdc25a ubiquitination). DUB3 can interfere with this process also inhibiting ubiquitin flagging of Cdc25a. During mitosis, Cdc25 protein is stabilized by the complex Cyclin-B/Cdk1 through phosphorylation. This post-translational stabilization is negatively controlled by the Wee1 kinase, which can inhibit Cyclin-B/Cdk1 activity.

**Figure 4:**
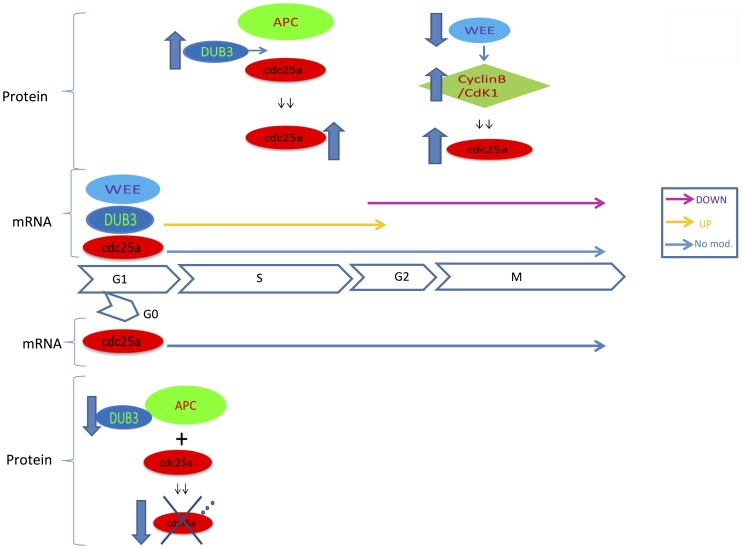
Transcriptional and post-translational modulation of CDC25A, DUB3 and Wee1 during cell cycle in PTEN active cell lines. CDC25A mRNA is constantly produced throughout all phases while DUB3 expression increases in the G1/S phase and wee1 expression decreases during the phase M. CDC25A modulation occurs at post translational level with DUB3 inhibiting its ubiquitination and Wee1 controlling negatively the activity of Cyclin-B/Cdk1.

By comparing GSCs to the differentiated counterparts, we observed that transcripts levels of Cdc25a, APC protein complex, SCF protein complex and Cyclin-B/Cdk1 do not vary after differentiation. Conversely, DUB3 mRNA is strongly upmodulated in differentiation and this could promote cell cycle progression, with the stabilization of Cdc25a, even in presence of DNA damage. Coherently, Wee1 appears to be one of the most downregulated genes, suggesting the release of Cyclin-B/Cdk1 from its inhibitory control, thus triggering the activation of Cdc25a in mitosis. Furthermore, it is interesting to observe that the mature form of miR-21 appears to be one of the RNAs that significantly fade during differentiation. miR-21 has been observed to target directly cdc25a in human cancer cells [26]. As above mentioned, Cdc25a mRNA levels are not influenced by the induction, while a post-transcriptional regulation, operated by a microRNA, should be reflected on its target transcript, at least partially. If the suggested post-transcriptional mechanism is actually involved in cell cycle regulation in GSCs, it probably occurs through the inhibition of translation instead of target mRNA degradation. A description of the involved actors and their variations through the induction of differentiation in GSC is schematized in figure 4.

According to that observed in PTEN positive GSCs, we propose a model in which Cdc25a is persistently transcribed during each of the cell cycle phases. In GSCs, its corresponding protein level is negatively regulated by its post-translational regulators, which contribute to maintain cells in interphase. The initial phase of the commitment to differentiation is the release from cell cycle progression control by the inhibition of cdc25a degradation by the activity of the augmented concentration of Dub3. This protein counteracts both the effects of APC/Cdh1 and of ubiquitination triggered by DNA damage checkpoints, providing cells the ability to progress through S and G2 phases. The stabilization of cdc25a through G2 and mitosis is also permitted by the diminished inhibitory effect of Wee1, which is strongly downregulated in differentiation, on Cyclin-B/Cdk1 complex. The downmodulation of miR-21 likely contributes also to the overall increase in cdc25a protein level by removing the post-transcriptional inhibition based on translation efficiency.

In this model, Dub3 and Wee1 are involved in cell cycle regulation in a cellular phenotype specific way. Dub3 promotes cell cycle progression in committed cells, although its transcript level remains low in stem cells. Conversely, Wee1 resulted expressed in stem cells, suggesting that its activity provide the ability to avoid G2/M transition.

While recent literature [24], [27] indicates Dub3 as an eligible therapeutic target for cancers expressing Cdc25a, our results imply that its inhibition may be ineffective in tumor initiating cells, which actually don't rely on Dub3 function. Moreover, therapeutic agents that can influence proliferation or survival of differentiated tumor cells may actually act in positive selection of GSCs from the tumor mass and remain ineffective in targeting cells that are able to promote neoplastic diffusion and disease recurrence. For this reason a deep understanding of molecular events underlying cell cycle regulation in PTEN-positive GSCs is fundamental in order to develop new effective therapies for glioblastoma treatment.

## Supporting Information

Figure S1 **Hierarchical clustering of samples according to their mean PTEN, AKT and pAKT protein expression levels.**
(TIFF)

File S1 **Table reporting whole genome transcriptomic modulation. Each column report samples's fold change along differentiation.**
(XLSX)
