# Supplementary figures and images for: Gene Expression Analysis of PTEN Positive Glioblastoma Stem Cells Identifies DUB3 and Wee1 Modulation in a Cell Differentiation Model
Source: PLoS One. 2013 Dec 12;8(12):e81432. doi: 10.1371/journal.pone.0081432 (PMC3861258; doi:10.1371/journal.pone.0081432)

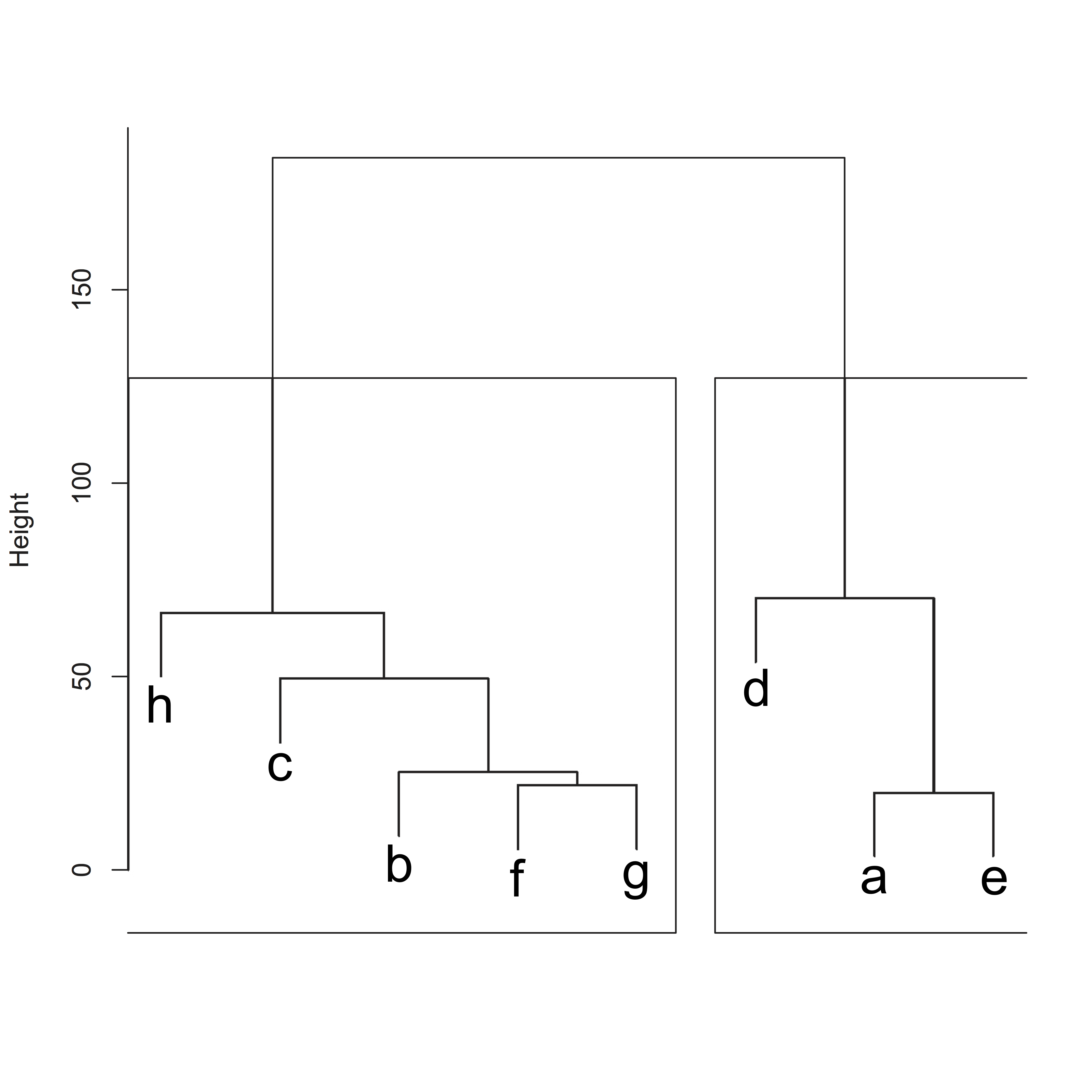

Supplement: Figure S1 — Hierarchical clustering of samples according to their mean PTEN, AKT and pAKT protein expression levels. (TIFF) [file pone.0081432.s001.tiff]
